# Glucose Transport and Transporters in the Endomembranes

**DOI:** 10.3390/ijms20235898

**Published:** 2019-11-24

**Authors:** Beáta Lizák, András Szarka, Yejin Kim, Kyu-sung Choi, Csilla E. Németh, Paola Marcolongo, Angelo Benedetti, Gábor Bánhegyi, Éva Margittai

**Affiliations:** 1 Department of Medical Chemistry, Molecular Biology and Pathobiochemistry, Semmelweis University, 1094 Budapest, Hungary; lizak.beata@med.semmelweis-univ.hu (B.L.); nemeth.csilla@med.semmelweis-univ.hu (C.E.N.); banhegyi.gabor@med.semmelweis-univ.hu (G.B.); 2 Laboratory of Biochemistry and Molecular Biology, Department of Applied Biotechnology and Food Science, Budapest University of Technology and Economics, 1111 Budapest, Hungary; szarka@mail.bme.hu; 3 Institute of Translational Medicine, Semmelweis University, 1094 Budapest, Hungary; dpwlsl487@gmail.com (Y.K.); albertchoi85@gmail.com (K.-s.C.); 4 Department of Molecular and Developmental Medicine, University of Siena, 53100 Siena, Italy; paola.marcolongo@unisi.it (P.M.); benedetti@unisi.it (A.B.)

**Keywords:** glucose, transporter, GLUT, SGLT, endomembrane

## Abstract

Glucose is a basic nutrient in most of the creatures; its transport through biological membranes is an absolute requirement of life. This role is fulfilled by glucose transporters, mediating the transport of glucose by facilitated diffusion or by secondary active transport. GLUT (glucose transporter) or SLC2A (Solute carrier 2A) families represent the main glucose transporters in mammalian cells, originally described as plasma membrane transporters. Glucose transport through intracellular membranes has not been elucidated yet; however, glucose is formed in the lumen of various organelles. The glucose-6-phosphatase system catalyzing the last common step of gluconeogenesis and glycogenolysis generates glucose within the lumen of the endoplasmic reticulum. Posttranslational processing of the oligosaccharide moiety of glycoproteins also results in intraluminal glucose formation in the endoplasmic reticulum (ER) and Golgi. Autophagic degradation of polysaccharides, glycoproteins, and glycolipids leads to glucose accumulation in lysosomes. Despite the obvious necessity, the mechanism of glucose transport and the molecular nature of mediating proteins in the endomembranes have been hardly elucidated for the last few years. However, recent studies revealed the intracellular localization and functional features of some glucose transporters; the aim of the present paper was to summarize the collected knowledge.

## 1. Introduction

The continuous supply of energy is an absolute requirement for maintaining metabolism, homeostasis, cell growth, and development. In heterotrophic organisms, energy is generated at the expense of nutrients, connecting their catabolism with the synthesis of high energy compounds, such as ATP. Glucose plays an essential role as an energy source in most of the organisms; therefore, how it passes through biological membranes is a fundamental question in biology. This process is achieved by facilitated diffusion or secondary active transport mediated by glucose transporters, a wide variety of membrane proteins [1]. These proteins have been primarily characterized as plasma membrane transporters mediating the uptake of glucose, other hexoses, and some related compounds, such as myoinositol or dehydroascorbic acid (DHA) [2]. Glucose uptake into and release from intracellular organelles have not attracted much interest. However, recent advances in the field of the compartmentalization of intermediary metabolism revealed that glucose is formed in the lumen of various organelles, which necessitates the presence of glucose transporters in endomembranes. For instance, the last shared reaction of gluconeogenesis and glycogenolysis is the hydrolysis of glucose-6-phosphate to free glucose and inorganic phosphate catalyzed within the lumen of the endoplasmic reticulum (ER) [3]. Intraluminal glucose formation in the ER and Golgi also accompanies the posttranslational maturation of oligosaccharide moiety of glycoproteins [4]. Polysaccharide, glycoprotein, and glycolipid breakdown during autophagy results in glucose accumulation in lysosomes [5]. Even though the aforementioned processes undoubtedly require glucose transport through the membranes of intracellular organelles, the mechanism and identity of such mediating proteins have been hardly elucidated for the last few years. However, the intracellular localization and functional characteristics of some glucose transporters have been recently described; the aim of this review was to collect the progress made in the field.

## 2. Glucose Transporters

Two families of glucose transporters have been identified in humans, including the major glucose facilitator GLUT (glucose transporter) superfamily (solute carrier 2A(SLC2A)) and the sodium-driven glucose symporter SGLT (sodium-glucose cotransporter) family (SLC5). Besides these classical transporter families, other proteins can also be involved in sugar transport, like the recently identified SWEETs (SLC50) and members of the Spinster (SLC63) protein family. 

### 2.1. SGLTs

The human sodium-glucose cotransporter family (SLC5) has 12 human genes expressed in various tissues. The corresponding membrane proteins mainly function as tightly coupled plasma membrane Na(+)/substrate cotransporters for solutes, such as glucose, myoinositol, and anions (including short-chain fatty acids); however, the family includes a Na(+)/Cl(−)/choline cotransporter and a glucose activated ion channel. SGLT1 and SGLT2 (SLC5A1 and A2) have a prominent role in glucose uptake through the plasma membrane. The members of the family mediate a secondary active transport by using the sodium concentration gradient between the two sides of the membrane. They are symporters; the stoichiometric ratios of sodium coupling of SGLT1 and SGLT2 have been characterized to be 2:1 and 1:1, respectively. They can facilitate glucose uptake even against a concentration gradient; therefore, they are present in locations where glucose absorption or reabsorption occurs; SGLT1 is primarily expressed in the intestine, while SGLT2 in the kidney [6].

The other members of the SGLT family seem to fulfill functions other than glucose transport. SGLT3 (SLC5A4) is a putative glucose sensor in different tissues [7]. SGLT4 (SLC5A9) and SGLT5 (SLC5A10) exhibit high affinity towards mannose, thus they can function as mannose transporters [8,9]. SGLT6 (SLC5A11) *alias* SMIT2 (sodium-myoinositol-cotransporter 2) mediates inositol transport [10].

### 2.2. GLUTs

The human facilitative glucose transporter family (GLUT or SLC2A) contains 14 isoforms with shared structural features, such as 12 transmembrane domains, N- and C-termini facing the cytoplasm of the cell, and N-glycosylation sites. Based on their sequence homology, they can be categorized into three classes: class I includes GLUT1-4 (SLC2A1-4) and GLUT14 (SLC2A14), class II the “odd transporters” GLUT5, 7, 9, 11 (SLC2A5, 7, 9, 11), and class III the “even transporters” GLUT6, 8, 10, 12, and 13 (SLC2A6, 8, 10, 12, 13). All members of the GLUT family are facilitative transporters with one exemption, the GLUT13, which is a proton-driven myoinositol transporter (also called human myoinositol transporter, or HMIT). The substrate specificity of these carriers is highly variable; they can mediate transmembrane fluxes of different hexoses, myoinositol, urate, glucosamine, and ascorbic acid (AA) [2]. However, the predominant substrate of all the GLUT carriers has not been described completely. The different isoforms show different tissue distribution, subcellular localization, substrate binding affinities, and regulation [11]. Class II and III isoforms have been cloned and characterized more recently; hence, their physiological role remains unclear. Class III GLUT transporters have an intracellular retention signal, making them good candidates for endomembrane sugar transport. 

### 2.3. Others

Besides the classic glucose transporter families, recent findings demonstrated the existence of other glucose transporters. The new SWEET class of glucose uniporters (SLC50) emerged as sugar efflux transporters; they are present mostly in plants. This family of transporters is represented by a single member, SWEET1 (or SLC50A1), in the human genome. While sugar efflux mediated by plant isoforms is induced by bacterial symbionts and different pathogens indicating that it serves the nutritional supply for pathogens and symbionts, the animal homologs are probably involved in sugar efflux from glucose producing (gluconeogenic) cells, such as intestinal, liver, epididymal, and mammary cells [12].

The Spinster (SLC63) gene family encodes evolutionarily conserved proteins belonging to the major facilitator superfamily. Drosophila contains one, and mammals carry three Spns homologs Spns1 (SLC63A1), Spns2 (SLC63A2), and Spns3 (SLC63A3) [13]. The fruit fly spin and mammalian Spsn1 seems to be involved in sugar export from lysosomes, Spns2 is a putative spingosine-1-phosphate (or sphingolipid) transporter, while the functions of Spns3 have not been clarified yet [13].

## 3. Processes Linked to Glucose Transport in the Organelles

### 3.1. Glucose Production by Glucose-6-Phosphatases

**Figure 1:**
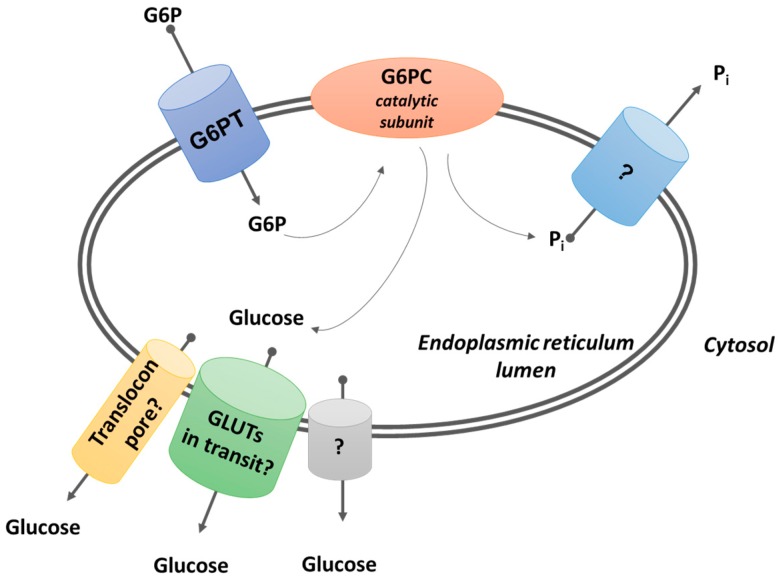
G6PC (glucose-6-phosphatase) is a transmembrane enzyme with the catalytic subunit facing the endoplasmic reticulum (ER) lumen. It drives the hydrolysis of glucose-6-phosphate (G6P) to glucose and inorganic phosphate (P_i_). The enzyme compartmentation requires transporters for G6P, Pi, and glucose. The identity of Pi transporter has not been clarified yet. Three possible transport mechanisms may explain the exit of glucose from the ER: translocon pore, glucose transporter (GLUT) transporters in transit through the secretory pathway, or a yet unidentified glucose transporter.

Glucose-6-phosphatase (G6Pase) is a transmembrane enzyme with the catalytic subunit located in the lumen of the ER network, and it is involved in driving the hydrolysis of glucose-6-phosphate (G6P) to glucose and inorganic phosphate (Pi) [3] (Figure 1). The enzyme compartmentation is a condition that requires G6P transport in the ER to allow its hydrolysis. This substrate is imported into the ER by a G6P-transporter (G6PT or SLC37A4) encoded by the *SLC37A4* gene. The human G6PT is a 46 kDa protein [14] encoded by a single copy gene mapped to chromosome 11q23 [15] and containing nine exons [16,17,18]. 

#### 3.1.1. Glucose-6-Phosphatases

In humans, there are at least three G6Pase enzymes encoded by different genes and with unique roles, tissue distribution, and kinetic properties. The classic G6Pase, also termed G6PC1 or G6Pase, is expressed in the liver, kidney, and small intestine [3,19]. It is a 35 kDa protein encoded by the *G6PC* single-copy gene that consists of five exons. The translated G6PC1 is a highly hydrophobic protein deeply inserted in the ER membrane [20]. It is well known that G6PC1 efficiently hydrolyzes G6P derived from glycogenolysis or gluconeogenic precursors and, therefore, plays an essential role in blood glucose homeostasis. Clear evidence of this role is a metabolic disease called type 1a glycogen storage disease (GSD 1a) or von Gierke disease, characterized by Cori and Cori in 1952 [21], that is caused by G6PC1 activity deficit leading to glycogen accumulation and characterized by poor tolerance to fasting, growth retardation, and hepatomegaly as a result of the accumulation of glycogen and fat in the liver. A variant of this metabolic disorder is caused by G6PT transporter’s functional deficit, leading to impaired transport of G6P in the ER and, therefore, to lower enzyme activity, being G6PC1 and G6PT functionally coupled. This condition is called type 1b glycogen storage disease and, in addition to the clinical profile shared with type 1a, it is characterized by neutropenia and altered neutrophil function predisposing to recurrent bacterial infections [22,23]. 

Traditionally, two further subtypes of GSD had been hypothesized, namely, GSD 1c and 1d, and caused, respectively, by a deficit in Pi or glucose transport across the ER membrane [22,24]. However, up to date, all the non-a GSD phenotypes have been shown to bear mutations in the G6PT gene and are not genotypically different pathologic conditions with a clear cut defect in the Pi or glucose transport function [25,26].

The G6PC2 is expressed mainly in the pancreatic beta-cells and was originally called “islet-specific G6PC related protein”, IGRP [3]. It is a 40.7 kDa protein encoded by the *G6PC* single-copy gene mapped to chromosome 2q31 that consists of five exons. The role of G6PC2 has been clarified from studies on *G6pc2*−/− mice strains [27]. The pathophysiological role of G6PC2 is less known. On the basis of experimental results and human genome-wide association studies, G6PC2 appears to play a role as a “negative” glucose sensor in pancreatic islets [3].

G6PC3 is ubiquitously expressed [3]. The human, single-copy *G6PC3* gene consists of six exons on chromosome 17q21 and encodes for a 38.7 kDa protein.

As to G6PC3, its deficiency is implicated in the severe congenital neutropenia syndrome type 4 (SCN4) that is not a real glycogen storage disease but can be considered a glycogen storage disease 1 related syndrome (GSD1rs). G6PC3 deficit shares neutropenia and neutrophil function defects typical to GSD1b [3]. G6PC3 is relevant to maintain macrophage/neutrophil homeostasis, though the exact mechanism has been elusive for a long period. It has been proposed that a dysfunctional G6PC3/G6PT complex may be related to the problem. G6PC3—similar to G6PC1—needs G6P supply in the ER lumen; thus, if the complex doesn’t work properly, the hydrolysis cannot work either. It has been evidenced that in GSD1b patients, neutrophils are more prone to apoptosis [28]. Neutrophils need glucose, and the destruction of G6PC3/G6PT complex alters glucose recycling in the ER. Thus, disturbed glucose homeostasis leads to neutropenia and neutrophil dysfunction. Nonetheless, also monocyte/macrophages are affected [29]. It is also possible that Hypoxia Inducible Factor HIF-1alpha is involved in the process [30]. Eventually, defective glucose production in the ER can be the cause of aberrant glycosylation [31], leading to neutrophil functional derangement. However, a very recent study [32] indicated that this enzyme could efficiently hydrolyze and eliminate the non-classical toxic metabolite 1,5-anhydroglucitol, at least, in human leukocytes. These findings provided a logical explanation for the leukocyte pathology of patients with G6PC3 and G6PT mutations and supported the hypothesis that G6PC3 would behave as a metabolite repair enzyme acting on substrates other than G6P [32].

#### 3.1.2. Possible Routes of Glucose Exit from the ER

On a functional basis, the route of exit of the produced Pi and glucose from the ER to the cytosol is still not completely clarified. It has been proposed that G6PT acts as a G6P/Pi antiporter through the ER membrane on the basis of experiments with the reconstituted protein in liposomes [33], but data obtained with liver microsomal vesicles led to the conclusion that G6PT is a facilitative G6P uniporter [34,35]. The exit of Pi from the ER is possibly allowed by transporters of the SLC37 family [36], though their biological functions have not been completely clarified, and their deficit has never been correlated to a Pi transport deficit phenotype.

On the other hand, transport of glucose across the ER membrane is of special relevance, at least, in the liver, kidney, and small intestine since glucose produced from G6P in the ER lumen, by G6Pase activity, must be exported to the cytosol and, hence, to the bloodstream. 

Liver microsomes are permeable to glucose [37,38], and two bidirectional facilitative transport systems appear to be operative, possibly connected to two ER glucose pools [39]. 

The glucose fluxes across ER membranes were also investigated in hepatoma cells (Hep G2) by using genetically encoded nanosensors for glucose targeted to the cytosol and ER compartments [40]. This study provided direct evidence for the presence of a high-capacity glucose transport system for import into and export from the intact ER network. It has been also proposed an alternative route of exit for ER glucose directly into the blood mediated by a vesicular efflux pathway [41]. This possibility, however, can hardly explain the high rate of glucose efflux from the liver under the circumstances, such as the fasting status, as previously detailed elsewhere [19]. 

Theoretically, at least three hypotheses may explain glucose efflux from the ER compartment: (i) an elusive transporter, responsible specifically for ER glucose export, does exist; (ii) in the ER membranes, there is a pore-like structure permeable to small uncharged molecules, including glucose [42]; (iii) plasma membrane glucose transporters of the GLUT family, during their transit to the plasma membrane, can mediate glucose efflux from the ER [43]. 

The lack of mutations in a gene corresponding to the glucose transport function [22,33] is not consistent with the existence of a unique glucose transporter (see hypothesis i) but states for the redundancy of transporters, though the lethality of mutations in the encoding gene for the glucose ER transporter is a possibility [43]; indeed, different transport systems can be evidenced at a functional level [38,39]. 

The possible role of pore-like structures (see hypothesis ii) accounting for ER glucose permeability has been investigated, and it has been shown that ER membranes can be permeable to small molecules [42], including small charged molecules that can pass through the translocon pore in the open status [44,45]. The translocon pore is likely involved in glucose transport but can’t be the only pathway for glucose ER efflux as the translocon is mainly associated with the rough ER, whereas G6Pase/G6PT are mainly expressed in smooth ER that typically lacks translocon [44]. This is especially true for the liver, the main organ expressing G6PC1 and involved in glucose homeostasis; it should be also observed that experimental evidence for a non-specific pore-like structure has been obtained in pancreatic microsomes that are highly enriched in rough ER [42].

Plasma membrane glucose transporters of the GLUT family (see iii), during their transit to the plasma membrane, can mediate glucose efflux from the ER [43]. Taking advantage of FRET-based nanosensors [43], the role of GLUT uniporters located in the ER during their way to the Golgi in glucose efflux was evaluated. Liver cells express different GLUTs [46]. The cooperation of different GLUT transporters, including GLUT 10 [47], in sustaining glucose efflux from the ER is possibly the reason for the redundancy of the transport function and the absence in human pathology of a clear phenotype of glucose efflux deficiency. Indeed, the only evidence of glucose accumulation in the ER is linked to GLUT2 deficiency (Fanconi–Bickel syndrome) [48]. GLUT 2 is the main isoform expressed in the liver and is involved in plasma membrane glucose transport and, eventually, in ER glucose efflux during the translocation towards the plasma membrane. GLUT 10 is an ER transporter [47] expressed in different tissues, including the liver. Its mutations lead to arterial tortuosity syndrome (ATS), which is possibly correlated to a defect of DHA transport in connective tissues [47] and does not affect glucose homeostasis. 

### 3.2. Protein Glycosylation/Deglycosylation

Protein glycosylation is one of the most common posttranslational modifications in every organism, having a crucial role in quality control and targeting through the secretory pathway [49]. Sugar addition to amino acid residues of the nascent polypeptide chain is initiated in the ER lumen and goes through further maturation in the Golgi network. N-glycans bear asparagine-linked oligosaccharide moiety, whereas O-glycans contain Ser or Thr-linked sugars [4]. 

**Figure 2:**
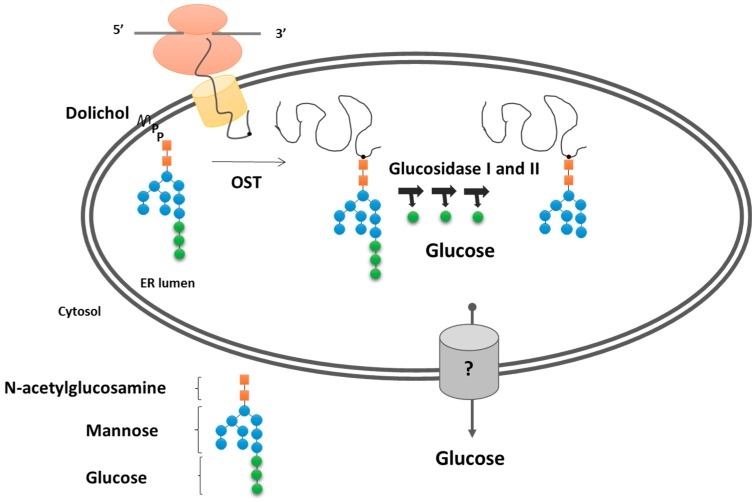
Protein glycosylation/deglycosylation in the endoplasmic reticulum. Oligosaccharyl-transferases link glycan moiety to nascent polypeptide chains. In the further maturation steps, glucosidase I and II trim the terminal glucose from oligosaccharide residue, and the free glucose is carried out of the endoplasmic reticulum via yet un-identified transporters. ER, endoplasmic reticulum; OST, oligosaccharyl-transferases.

N-glycosylation has been proposed as the most important controlling process in protein folding. The oligosaccharide chain has a well-defined structure and serves as a tag for sugar-binding chaperones, called lectins. The composition of sugars defines the folding state of the maturing polypeptide and regulates its fate. The oligosaccharide moiety is synthesized in a multiple-step process on a lipid linker, dolichol-phosphate on the cytosolic face of the ER membrane. The addition of the last sugars happens in the lumen, and the assembled GlcNAc_2_Man_9_Glc_3_ oligosaccharide is linked to the target proteins by oligosaccharyl-transferases (OST). The editing starts with the removal of the outermost glucose by glucosidase I and II, producing free glucose in the ER lumen. The resulting sugar structure would serve as a binding site for calnexin/calreticulin and guide the polypeptide to folding cycles. The last free glucose is cleaved by glucosidase II and targets the protein to further stations of the secretory pathway [50] (Figure 2).

O-glycosylation also starts in the ER lumen and serves as a starting point for the additional sugar linking in the assembly of proteoglycans and glycoproteins, the steps which later take place in the Golgi network. The maturation of the oligosaccharide moiety requires the addition and cleavage of sugars; thus, the Golgi is equipped with glucosidases and glucose-transferases. The glucosidase reaction again will result in the deliberation of free glucose [4]. 

How the glucose is exported from the ER and Golgi network remains unknown. A hypothesis for the ER glucose export has been proposed regarding the G6Pase system (see Section 3.1.2). Possible Routes of Glucose Exit from the ER. Some of the speculations would correspond to the Golgi situations as well, although translocon pores, for instance, do not exist in Golgi. GLUT transporters, trafficking through the secretory pathway, can mediate glucose export [43]. The vesicular transport and clearing the luminal content via exocytosis is also a possibility. However, some studies showed the function of some GLUT transporters in the *cis*-Golgi membrane, supplying substrates for lactose synthesis in the mammary gland [51]. 

### 3.3. Autophagy

Autophagy is the cellular process for the degradation and recycling of the cellular material in certain metabolic circumstances [5]. During this pathway, autophagosomes fuse with lysosomes, where their engulfed substances are hydrolyzed by lysosomal enzymes [52]. The hydrolytic degradation of sugar-containing macromolecules, like polysaccharides, glycoproteins, and glycolipids, leads to monosaccharide formation in the lumen of autolysosomes [53]. Lysosomal membranes are equipped with lysosomal efflux transporters to translocate these nutrients to the cytosol, although these processes are not completely described [54]. Abnormal lysosomal degradation or abnormal export of degraded products can lead to the accumulation of undigested materials in the lysosomal lumen, which is also observed in many neurodegenerative diseases [55].

**Figure 3:**
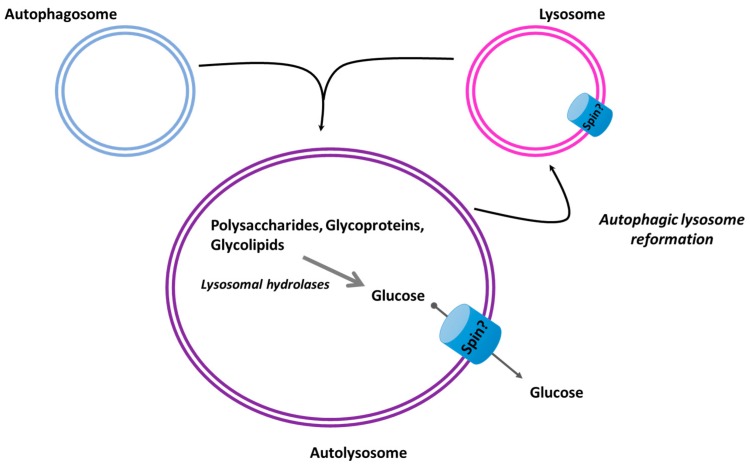
Sugar-containing macromolecules digested by lysosomal hydrolases. The resulting monosaccharides are exported from the autolysosomal lumen through possible sugar transporter Spin. Spin is also involved in autophagic lysosome reformation.

Free lysosomes are recovered after autophagy is terminated through autophagic lysosome reformation (ALR) [56]. This evolutionarily conserved process is regulated by the reactivated mTOR (mammalian target of rapamycin) kinases, which generates proto lysosomal-tubules and vesicles formed from the autolysosomal membrane, and mature into functional lysosomes [56]. The lysosomal sugar efflux transporter Spns was shown to be crucial for ALR. Mammalian and *Drosophila* cells lacking *spns* exhibited the accumulation of enlarged autolysosomes with substrate degradation defects. Amino acid residues essential for sugar transport activity of Spns were demonstrated to be indispensable for their function in lysosome reformation [57] (Figure 3).

The human ortholog HSpns1, overexpressed in cultured human cells interacted with Bcl-2/Bcl-xL and induced caspase-independent autophagic cell death [58]. An in vivo study using *spns1* mutant zebrafish detected excessive autophagosome and autolysosome accumulation, concomitant with impaired lysosomal acidification. The same study found that lack of Spns1 caused embryonic senescence, which was mitigated by Beclin 1 suppression and worsened by p53 suppression [59]. Further investigation of the role of Spns1 in zebrafish found that a concurrent disruption of the vacuolar-type H+-ATPase (v-ATPase) subunit gene, atp6v0ca (ATPase, H+ transporting, lysosomal, V0 subunit ca) led to suppression of the senescence induced by the Spns1 defect, but didn’t suppress the aberrant autolysosomal functions [60]. 

In a mouse model of lysosomal storage disease (Niemann–Pick type C disease-1), abnormal autophagy was observed, which could be ameliorated by the overexpression of Spns1 [61]. 

Taken the lysosomal efflux transporter, Spin1 clearly contributes to normal lysosomal homeostasis and functional autophagy pathway, although its exact role still needs to be investigated. Some of the members of the GLUT family were also found to be localized in the lysosomal membrane [62,63], but their functional role in this organelle and contribution of autophagy remains to be investigated.

## 4. In Silico Predictions of the Localization of Different Glucose Transporters

There is an easily acceptable requirement to know the intracellular localization of different glucose transport proteins. The known localization can contribute to a better understanding of the metabolic and regulatory pathways occurring in the given subcellular organelle. Several high-throughput experimental methods have been developed for the determination of the subcellular localization of proteins in the last two decades. Probably the most well-known is the green fluorescent protein that gives the localization of the generated fusion proteins through fluorescence screening [64]. Although it gives the possibility to follow the localization of the protein in vivo, the presence of the fusion/tagging protein may interfere with the sequence or structural signals necessary to target the protein of interest to its proper organelle. Another main line of the determination of subcellular localization is the homogenization and fractionation of the cell by differential and gradient centrifugation and then the identification of proteins in the various fractions by mass spectrometry or immunoblotting techniques [65]. The disadvantage of the latter method is the sensitivity to contaminations. In general, all the above mentioned high-throughput experimental techniques produce some false positive and false negative assignments.

To bride these pitfalls, several computational tools have been developed to score the likelihood that a protein belongs to a given compartment and complement the use of experimental methods [66]. Albeit these in silico prediction tools also have limitations, the scores provided by them can be used to improve the quality of high-throughput data and predict the localization of compartment-specific proteins [67]. Unfortunately, no comprehensive in silico analysis on the subcellular localization of the known glucose transporters could be found. Thus, as an additional tool to predict the subcellular localization of glucose transporters, the subcellular localization of the whole GLUT, SGLT, SWEET, and Spns families was investigated by three different computational prediction tools in this paper. In a previous study, our research group made an attempt to predict the possibility of the mitochondrial localization of the GLUT family members [67], while, in another, the subcellular localization of GLUT10 was analyzed by in silico prediction tools [47]. However, the first comprehensive in silico analysis of the subcellular localization of the known glucose transporters is presented here (Table 1, Table 2, Table 3 and Table 4). 

Our previous observations [67] strengthened the statement of Sprenger et al. (2006), who compared different prediction tools, that no individual method had a sufficient level of sensitivity across both evaluation sets that would enable the reliable application to hypothetical proteins. Thus, we decided to use three different prediction tools with different signal prediction methods. Moreover, prediction tools were chosen to use different algorithms. PSORTII uses the *k*-nearest-neighbor method to predict the subcellular localization of different proteins [68]. YLoc uses native Bayes alongside entropy-based discretization to make predictions [69]. YLoc was used in the high-resolution version to predict 11 eukaryotic subcellular locations and with gene ontology (GO) terms. CELLO is based on a two-level support vector machine (SVM) system: the first level comprises a number of SVM classifiers, each based on a specific type of feature vectors derived from sequences; the second level SVM classifier functions as the jury machine to generate the probability distribution of decisions for possible localizations [70]. We used the developed version of CELLO, the CELLO2GO, which provides brief and/or detailed annotations of GO terms related to homologs of a query protein found by BLAST searching in combination with a CELLO-predicted subcellular localization(s) for the queried protein [70]. The sequences of all members, including the known isoforms of both families, were extracted from SWISS-PROT.

Not surprisingly, the plasma membrane got the highest prediction localization score in the case of all GLUT family members (Table 1). 

Interestingly, our earlier prediction study resulted in different localization probability [67]. The mitochondrial localization of the whole GLUT family was examined earlier by eight different localization tools. The mitochondrial localization of GLUT 1, 9, and 11 got significant probability scores. However, in the previous studies, only the first 60 amino acid residues at the N-terminal were used for prediction analysis [67,71], as it was suggested that the length of reported mitochondrial targeting peptides spanned from six residues up to approximately 60 residues. Furthermore, the first study of in silico prediction tools also used the first 60 N-terminal amino acids for the subcellular localization analysis [71]. On the contrary, in our current study, the whole polypeptide sequence was analyzed, which might result in a different localization probability. Albeit the use of the whole amino acid sequence seems to be more precise, it is worth to note that the presence of GLUT1 in the mitochondrial inner membrane was also reinforced by different experimental methods [71].

The plasma membrane localization of the SGLT family (Table 2) is in concordance with their known experimental localization and physiological function [72].

Our in silico analysis of the localization of the novel Spns family (Table 3) did not reinforce their supposed lysosomal occurrence [13]. Necessarily, further observations need to determine their more exact subcellular localization.

The situation of the only known member of the human SWEET family is similar. Albeit PSORTII gave a high probability score for its localization in the ER membrane, other prediction tools suggested that it was located in the plasma membrane (Table 4).

## 5. Glucose Transport in the Endomembranes

Class III GLUT transporters (GLUT6, 8, 10, 12, 13) have a remarkable common structural feature; they all contain an internalization signal (dileucine or YSRI in case of GLUT10) [2]. These residues are responsible for keeping the transporters in intracellular membranes, but in response to certain stimuli or signal transduction events, the transporters can translocate to the plasma membrane. Similar to the extensively investigated GLUT4 plasma membrane translocation pathway, such a pathway is also under interest in the case of Class III GLUTs. In some cases, the cell surface appearance of the transporter has been described, but, in other cases, the transporter has been only detected intracellularly. GLUT12 has been demonstrated to translocate to the plasma membrane of muscle cells in a pathway similar to GLUT4 [73]. GLUT13 has been proved to be H^+^ coupled myoinositol transporter [74]. However, there are several experimental data implying that GLUT6, 8, and 10 are possible candidates to serve as monosaccharide carriers through endomembranes.

Spsn1 from the Spinster gene family also seems to function as a lysosomal sugar efflux transporter [13]. 

### 5.1. GLUT6

GLUT6 (SLC2A6, formerly referred to as GLUT9) was first cloned from human leukocytes using the conserved ‘hexose transporter signatures’ in expressed sequence tag (EST) databases [75]. It was found to be expressed mainly in the brain, spleen, and peripheral leucocytes. Hexose transport for GLUT6 was demonstrated in reconstituted liposomes, where GLUT6 transport activity was found only in the presence of 5 mM but not 1 mM glucose and exhibited a low cytochalasin B binding affinity [75].

GLUT6 carries the N-terminal dileucine motif targeting the transporter to intracellular membranes. Expression of the HA-tagged GLUT6 revealed that this protein only appeared on the cell surface when the dileucine residues were mutated to alanine or when endocytosis was blocked by the expression of a dominant-negative dynamin. In the same study, various stimuli were experimented to provoke plasma membrane translocation of GLUT6, but all of them failed. They concluded that GLUT6 seemed to be recycling between the plasma membrane and endomembranes, but its physiological site of function was unclear [76]. The relatively low in silico plasma membrane localization score of GLUT6 also supported its possible subcellular localization (Table 1).

GLUT6 was further found to be among the most upregulated genes in different tumor cell lines, like chronic lymphocytic leukemia [77] and malignant endometrium [78]. Abolishing of GLUT6 expression inhibited glycolysis and survival of endometrial cancer cells [78]. GLUT6 was also upregulated in LPS (lipopolysaccharide) stimulated macrophages in an NF-kB (Nuclear factor kappa B) dependent way. In this study, GLUT6 was demonstrated to localize to lysosomal membranes [63]. Although LPS-stimulated macrophages exhibit a high level of GLUT6 induction, suppression of GLUT6 expression did not alter the function of this cell line [79]. GLUT6 knockout mouse was also developed but did not show significant changes in whole-body physiology [80].

Many pieces of evidence show that GLUT6 might be an intracellular glucose transporter, with variable tissue-specific regulation. The exact cellular function of GLUT6 remains enigmatic.

### 5.2. GLUT8

GLUT8 was first discovered using EST database searches based on homology with conserved sugar transporter motifs [81,82]. GLUT8 is a member of the class III transporters bearing a characteristic large and presumably glycosylated extracellular loop 9 [83]. Its glucose transport activity showed high affinity (*K*_m_ 2 mM) and found to be inhibited by cytochalasin B and partly competed by D-fructose and D-galactose. Substrate specificity was further expanded by demonstrating DHA transport in GLUT8-expressing oocytes, which was inhibited by glucose, fructose, and by the flavonoids phloretin and quercetin [84].

GLUT8 was the first among hexose transporters implicated as a putative intracellular sugar carrier, with many experimental pieces of evidence detecting it in subcellular compartments [62]. The exact subcellular localization was examined in different tissues observing either endogenous or overexpressed GLUT8 proteins using a wide range of techniques. The results localized the transporter to various endomembranes: lysosomes, late endosomes, trans-Golgi, or the rough ER. The possible ER localization of GLUT8 was also supported by the 26.1% probability for ER localization given by the PSORTII computational tool (Table 1). N-terminal domain of GLUT8 contains a dileucine motif that was demonstrated to direct proteins to intracellular membranes and lysosomes [85,86], indeed mutation of the leucine residues to alanine changed the transporter localization to the plasma membrane [76,87]. GLUT4 having the same dileucine motif is well known for translocating to the plasma membrane upon insulin stimuli [88]. Similarly, other members of the class III glucose transporters also moved to the cell surface in response to various treatments [89,90]. The trigger for GLUT8 translocation was also tested in rat adipose cells [76] or murine neuronal cells [91,92], but all the treatments in these cell types failed to bring the transporter to the plasma membrane.

Immunohistochemical analyses of GLUT8 expression also proved its subcellular localization in human brain samples. Immunoreactivity of GLUT8 antibody was found in astrocytic and microglial cells in subependymal areas of human brains and also epithelial cells of the choroid plexus and ependymal cells [93].

In intestinal tissues, the intracellular localization of GLUT8 was also proved by immunohistochemistry studies showing a supranuclear distribution next to the apical membrane [94]. The investigation of the physiological role of GLUT8 in hexose uptake revealed that GLUT8 was an intracellular transporter that suppressed fructose uptake through the enterocytes. This study demonstrated that GLUT8-deficient mice exhibited increased enterocyte fructose absorption and increased serum fructose concentrations after oral fructose administration [95]. GLUT8 was also regulating the levels and trafficking of GLUT12. 

A subcellular role of GLUT8 was also demonstrated in the mammary gland to support lactose synthesis. GLUT8 was found to be upregulated during mammary gland progression. Endogenous GLUT8 and GLUT1 were localized to *cis*-Golgi in association with lactose synthase complex, suggesting that both transporters were involved in glucose uptake into this organelle [51].

Some studies, however, showed GLUT8 functioning in the plasma membrane in certain tissues, raising the possibility that this transporter has a tissue-specific regulation. First, GLUT8 was demonstrated to respond to insulin addition on blastocysts. The endogenous transporter translocated to the plasma membrane after insulin treatment [96]. Insulin-sensitivity of the transporter was also demonstrated in the heart, implying that it could compensate for the functions of GLUT4. This study reported that GLUT8 translocated from an intracellular pool to the cell surface of the healthy myocardium upon insulin stimulation, and lack of insulin (aka type I Diabetes) down-regulated the total protein expression and trafficking to the myocardial plasma membrane [97,98]. GLUT8 also seems to function as a cell surface transporter in the liver. Mice lacking GLUT8 developed impaired first-pass hepatic fructose metabolism, suggesting that fructose uptake by hepatocytes could be partly mediated by GLUT8. It was demonstrated that GLUT8 was a cell surface-localized transporter and that GLUT8 overexpression or GLUT8 shRNA-mediated gene silencing significantly induced or blocked radiolabeled fructose uptake in cultured hepatocytes [99]. GLUT8 deficiency could also prevent fructose-induced metabolic changes, such as hepatic macrosteatosis and triglyceride accumulation, suggesting its important role in hepatic fructose uptake. Trehalose is autophagy inducing disaccharide, known to alleviate symptoms of several neurodegenerative diseases, with a so-far unexplained mechanism. The hepatic entry of trehalose was also demonstrated to be mediated by GLUT8 [100].

### 5.3. GLUT10

GLUT10 is a member of the class III GLUT family and is encoded by the *SLC2A10* gene. The gene is located on the long arm of chromosome 20, in the *region* q12-13.1, and is organized into five exons and four introns [101,102]. So far, 35 mutations have been identified in the gene, including 21 missense substitutions, five nonsense nucleotide changes, eight deletions causing an early stop codon or frameshift, and a splice mutation [103,104,105,106,107,108,109,110,111,112,113]. Mapped mutations of GLUT10 result in ATS, a rare multi-organ connective tissue disorder. The human GLUT10 protein contains 541 amino acids with a predicted molecular mass of 57 kDa and shares some sequence similarity with GLUT 1-8 [102]. The secondary structure of GLUT10 comprises of 12 transmembrane helices, which are organized into two bundles [101,114]. The protein contains an intracellular loop between helices 6 and 7, and another loop is located extracellularly between helices 9 and 10, also including a potential N-linked glycosylation site [101]. Hydropathy analysis of the predicted protein shows structural similarity with other sugar transporters, although the size and location of the extracellular loop are distinctive between GLUT10 and the other family members [101].

The transport activity and the subcellular localization of GLUT10 have been a matter of debate for a long time. In *Xenopus* oocytes, after human GLUT10 mRNA injection, 2-deoxy-d-glucose transport activity could be observed, which competed with D-glucose or D-galactose. Phloretin—a general inhibitor of mammalian glucose transporters—was reported as a potent inhibitor of the transporter [102]. They haven’t analyzed the subcellular localization of the protein, but, based on the gene localization and functional properties, they suggested that GLUT10 might be involved in glucose metabolism and type 2 diabetes.

Lee and colleagues [115] found that under normal conditions, GLUT10 localized to Golgi in adipocytes, while insulin stimulated the translocation of the transporter into mitochondria. In mouse *AVSMCs* and rat A10 cells, GLUT10 was abundant in mitochondria, where it was shown to enhance DHA uptake. In H_2_O_2_-treated cells, DHA reduced the level of reactive oxygen species (ROS), protecting cells from ROS-induced vascular damage [115]. Later it was shown that human HEK293 cells express a functional AA transporter that coincides sodium coupled ascorbic acid transporter (SVCT2), which questioned the importance of GLUT10-dependent mitochondrial AA uptake [116]. In silico predictions disproved the role of GLUT10 in mitochondrial DHA transport, as the transporter achieved the lowest mitochondrial localization scores [67].

Additional studies described GLUT10 in the endomembrane system of the cell. Coucke et al. found GLUT10 protein of human fibroblasts in the perinuclear region [103], and Segade demonstrated that GLUT10 localized to the ER by using PAC-1 rat aortic smooth muscle cells too [117]. Regarding the transport activity, the paper questioned GLUT10 as a *bona fide* glucose transporter and suggested AA to be its physiological ligand. This would be in accordance with the observed symptoms of ATS patients, such as laxity of the skin and hyperextensibility of the joints, as in ATS, the decreased DHA uptake might result in insufficient levels of available AA for the posttranslational modification of extracellular matrix proteins in the ER [117]. Németh et al. showed that GLUT10 was indeed a DHA transporter of the ER, and DHA transport is defective through the endomembranes of ATS fibroblasts [47,118]. Its possible ER localization was also reinforced by the relatively high 39.1% of the ER prediction score of PSORTII (Table 1).

AA serves as a cofactor of numerous Fe^2+^/2-oxoglutarate-dependent dioxygenases and is hypothetically the key epigenetic regulator of gene expression in the nucleus [119]. Ascorbate-dependent enzymes of the Jumonji family catalyze histone demethylation [120], while those of TET (ten-eleven translocation) family support active demethylation via 5-methylcytosine hydroxylation [121]. GLUT10 being localized to the ER membrane or the nuclear envelope may contribute to physiological AA levels of the nucleus by transporting its oxidized form, DHA. DHA may be reduced to AA in the nucleus, which presumably contributes to the modification of the epigenetic pattern by altering the activity of ascorbate-dependent enzymes [122]. Indeed, it was found that AA increased the hydroxymethylation level of DNA only in control fibroblasts but not in cells deriving from ATS patients [123]. In accordance with this, transcriptome analysis revealed gene expression changes in ATS fibroblasts [124]. Increased DNA methylation can lead to changes in the gene expression of GLUT10 too. Novakovic’s group described that promoter methylation regulated expression of GLUT3 and GLUT10 in placental tissue [125].

### 5.4. Spns1

Spinster (Spns or benchwarmer) was described in Drosophila as a putative lysosomal efflux transporter with predicted sugar carrier amino acid sequence belonging to the major facilitator superfamily [126]. First, it was identified in genetic screens in mutant Drosophila lines, showing neurodegenerative phenotypes [127,128]. Spns was demonstrated to localize to the late endosomes/lysosomes in Drosophila [126], as well as its vertebrate homolog in zebrafish [129], and also its mammalian counterpart [57]. RFP or GFP-tagged Spns in fruit fly muscle tissue localized to an expansive tubular lysosomal network in live-cell imaging experiments and didn’t show co-localization with ER, mitochondrial, or Golgi markers [130]. Drosophila Spns mutants exhibited the accumulation of lysosomal carbohydrates and enlarged lysosomes [126], while, in mammalian cells, loss of Spns resulted in the accumulation of enlarged autolysosomes. Amino acid residues critical in sugar transporter activity of Spns were demonstrated to be crucial for ALR [57]. Co-localization of Spns1 with autophagosome markers was further demonstrated in other studies [60,130], suggesting the role of this lysosomal transporter in autophagy. The sugar carrier activity and the precise substrate specificity of Spns still need to be demonstrated since it is based on only structural predictions. 

## 6. Conclusions

Compartmentalization of eukaryotic cells ensures the optimal distribution of cellular tasks by separating different reactions to different membrane-enclosed organelles. Such a subcellular organization also requires the transmembrane flux of metabolites between compartments; thus, transport proteins of intracellular membranes are important contributors to cellular homeostasis. Subcellular sugar transport processes have not been in focus of interest before, although several well-known intraluminal reactions lead to the formation of glucose. Recent studies revealed some possible candidates for intracellular sugar transporters. Members of class III GLUT transporter family were shown to be presented in endomembranes, such as GLUT6 in lysosomes, GLUT 8 in lysosomes, ER or Golgi network, and GLUT10 in ER or nuclear envelope. Spns1 from another carrier family was also demonstrated to possibly contribute to lysosomal sugar efflux. It was also suggested that plasma membrane GLUT transporters while being carried through the secretory pathway, could fulfill sugar transporting duties. Further investigation is needed to explain the accurate role of diverse glucose transporters in subcellular organelles. Since proper metabolite export from compartments is also important in understanding glycogen storage or lysosomal storage diseases, more studies are expected to clarify this compartmentalization problem. 

## Figures and Tables

**Table 1:** In silico prediction of subcellular localization of human GLUT (glucose transporter) (SLC (solute carrier) 2) family. The sequences of all GLUT transport proteins were retrieved from the Uniprot database (http://www.uniprot.org/). ER (endoplasmic reticulum). The analyses were done on 3 October 2019.

Transporter	Location	PsortII	yLoc	Cello2GO
**Glut1**	Plasma membrane	65.2%	93.9%	4.937 (98.74%)
	ER	17.4%	0.2%	
	Vacuolar pathway	8.7%	0.2%	
	Peroxisome		5.9%	
	Cytoplasm			0.014 (0.28%)
	Nucleus			0.009 (0.18%)
**Glut2**	Plasma membrane	69.6%	86.3%	4.963 (99.26%)
	ER	26.1%	0.8%	
	Extracellular space		12.7%	
	Peroxisome			0.007 (0.14%)
	Mitochondrion	4.3%		
	Nucleus			0.007 (0.14%)
**Glut3**	Plasma membrane	78.3%	90.0%	4.930 (98.6%)
	ER	17.4%	0.5%	
	Extracellular space		9.4%	
	Lysosome			0.011 (0.22%)
	Peroxisome			0.026 (0.52%)
	Mitochondrion	4.3%		
**Glut4**	Plasma membrane	60.9%	88.3%	4.957 (99.14%)
	ER	17.4%		
	Extracellular space			0.011 (0.22%)
	Vacuolar	8.7%		
	Peroxisome		10.9%	0.005 (0.1%)
	Cytoplasm		0.4%	
**Glut5**	Plasma membrane	52.2%	53.6%	4.970 (99.4%)
	ER	34.8%		
	Extracellular space		1.9%	0.006 (0.12%)
	Peroxisome		43.7%	0.006 (0.12%)
	Mitochondrion	4.3%		
**Glut6**	Plasma membrane	60.9%	54.0%	4.970 (99.4%)
	ER	17.4%		
	Extracellular space		0.1%	
	Vacuolar	8.7%		
	Peroxisome		45.8%	
	Mitochondrion			0.009 (0.18%)
	Lysosome			0.009 (0.18%)
**Glut7**	Plasma membrane	60.9%	72.5%	4.943 (98.86%)
	ER	21.7%	0.1%	
	Vacuolar	8.7%		
	Peroxisome		27.4%	0.019 (0.38%)
	Mitochondrion			0.012 (0.24%)
**Glut8**	Plasma membrane	69.6%	98.8%	4.949 (98.98%)
	ER	26.1%		
	Peroxisome		1.0%	
	Mitochondrion	4.3%		0.008 (0.16%)
	Lysosome		0.1%	0.018 (0.36%)
**Glut9**	Plasma membrane	73.9%	2.0%	4.961 (99.22%)
	ER	21.7%		
	Peroxisome		94.1%	0.010 (0.2%)
	Mitochondrion	4.3%		
	Cytoplasm		3.2%	
	Lysosome			0.006 (0.12%)
**Glut10**	Plasma membrane	43.5%	99.8%	4.853 (97.06%)
	ER	39.1%	0.1%	
	Extracellular space		0.1%	0.061 (1.22%)
	Lysosome			0.028 (0.56%)
	Mitochondrion	4.3%		
**Glut11**	Plasma membrane	44.4%	75.1%	4.924 (98.48%)
	ER	55.6%	19.6%	
	Extracellular space		5.3%	
	Peroxisome			0.014 (0.28%)
	Mitochondrion			0.017 (0.34%)
**Glut12**	Plasma membrane	82.6%	45.3%	4.958 (99.16%)
	ER	17.4%	0.4%	
	Peroxisome		54.1%	
	Mitochondrion			0.005 (0.1%)
	Lysosome			0.005 (0.1%)
**Glut13**	Plasma membrane	65.2%	14.6%	4.879 (97.58%)
	ER	17.4%		
	Vacuolar	8.7%		0.023 (0.46%)
	Peroxisome		77.9%	
	Mitochondrion			0.016 (0.32%)
	Cytoplasm		5.2%	
**Glut14**	Plasma membrane	65.2%	99.5%	4.971 (99.42%)
	ER	26.1%		
	Extracellular space		0.4%	
	Peroxisome		0.1%	0.011 (0.22%)
	Nucleus	4.3%		
	Lysosome			0.004 (0.08%)

**Table 2:** In silico prediction of subcellular localization of human SGLT (Sodium glucose cotransporter) family. The sequences of all SGLT transport proteins were retrieved from the Uniprot database (http://www.uniprot.org/). The analyses were done on 3 October 2019.

Transporter	Location	PSORT II	yLoc	Cello
**SGLT1**	Plasma membrane	73.9%	99.2%	4.969 (99.38%)
	ER	13.0%	0.3%	0.004 (0.08%)
	Vacuolar	8.7%		
	Peroxisome		0.3%	
	Nucleus			0.005 (0.1%)
**SGLT2**	Plasma membrane	69.6%	99.8%	4.966 (99.32%)
	ER	13.0%		
	Vacuolar	8.7%		
	Peroxisome		0.1%	
	Mitochondrion			0.003 (0.06%)
	Lysosome			0.006 (0.12%)
**SGLT3**	Plasma membrane	69.6%	99.5%	4.981 (99.62%)
	ER	13.0%	0.3%	0.003 (0.06%)
	Vacuolar	8.7%		
	Lysosome		0.2%	0.002 (0.04%)
**SGLT4**	Plasma membrane	82.6%	99.9%	4.967 (99.34%)
	ER	13.0%		
	Peroxisome			0.004 (0.08%)
	Mitochondrion	4.3%		0.004 (0.08%)
**SGLT5**	Plasma membrane	69.6%	99.9%	4.968 (99.36%)
	ER	13.0%		
	Vacuolar	8.7%		
	Nucleus			0.007 (0.14%)
	Lysosome			0.008 (0.16%)

**Table 3:** In silico prediction of subcellular localization of human SPNS (Spinster homologue) family. The sequences of all SPNS transport proteins were retrieved from the Uniprot database (http://www.uniprot.org/). The analyses were done on 3 October 2019.

Transporter	Location	PSORT II	yLoc	Cello
**SPNS1**	Plasma membrane	73.9%	74.2%	4.970 (99.4%)
	ER	21.7%		
	Extracellular space			0.007 (0.14%)
	Peroxisome		25.3%	0.004 (0.08%)
	Mitochondrion	4.3%		
	Cytoplasm		0.3%	
**SPNS2**	Plasma membrane	60.9%	78.8%	4.897 (97.94%)
	ER	21.7%		
	Vacuolar	8.7%		
	Peroxisome		20.9%	0.016 (0.32%)
	Mitochondrion			0.023 (0.46%)
	Nucleus		0.3%	

**Table 4:** In silico prediction of subcellular localization of the human SWEET protein. The sequence of the human SWEET transport protein was retrieved from the Uniprot database (http://www.uniprot.org/). The analyses were done on 3 October 2019.

Transporter	Location	PSORT II	yLoc	Cello
**hSWEET1**	Plasma membrane	22.2%	80.6%	4.881 (97.62%)
	ER	33.3%		
	Extracellular space		18.4%	0.020 (0.4%)
	Vacuolar	22.2%		
	Nucleus			0.030 (0.6%)
	Lysosome		0.6%	

## Abbreviations

AA	Ascorbic acid
ALR	Autophagic lysosome reformation
ATS	Arterial tortuosity syndrome
DHA	Dehydroascorbic acid
EST	Expressed sequence tag
ER	Endoplasmic reticulum
HIF	Hypoxia Inducible factor
HMIT	Human myoinositol transporter
G6P	Glucose-6-phosphate
G6PT	Glucose-6-phosphate transporter
G6Pase	Glucose-6-phosphatase
G6PC	Glucose-6-phosphatase
GLUT	Glucose transporter
GO	Gene ontology
GSD	Glycogen storage disease
GSD1rs	Glycogen storage disease 1 related syndrome
LPS	Lipopolysaccharide
mTOR	Mammalian target of rapamycin
Nf-kB	Nuclear factor kappa B
OST	Oligosaccharyl-transferase
Pi	Inorganic phosphate
ROS	Reactive oxygen species
SCN4	Severe congenital neutropenia type 4
SGLT	Sodium-glucose cotransporter
SLC	Solute carrier
SMIT2	Sodium-myoinositol cotransporter 2
SPNS	Spinster homologue
SVCT	Sodium couplet ascorbic acid transporter
SVM	Support vector machine
TET	Ten-eleven translocation
